# A Predictive Study Between Anxiety and Fear of COVID-19 With Psychological Behavior Response: The Mediation Role of Perceived Stress

**DOI:** 10.3389/fpsyt.2022.851212

**Published:** 2022-03-22

**Authors:** Hamid Sharif Nia, Long She, Harpaljit Kaur, Christopher Boyle, Fatemeh Khoshnavay Fomani, Esmaeil Hoseinzadeh, Daniyal Kohestani, Pardis Rahmatpour

**Affiliations:** ^1^Amol Faculty of Nursing and Midwifery, Traditional and Complementary Medicine Research Center, Addiction Institute, Mazandaran University of Medical Sciences, Sari, Iran; ^2^School of Business, Swinburne University of Technology, Sarawak, Malaysia; ^3^Faculty of Business and Law, Taylor's University, Subang Jaya, Malaysia; ^4^School of Education, The University of Adelaide, Adelaide, SA, Australia; ^5^School of Nursing and Midwifery, Tehran University of Medical Sciences, Tehran, Iran; ^6^School of Nursing and Midwifery, Tehran Islamic Azad University of Medical Sciences, Tehran, Iran; ^7^School of Nursing and Midwifery, Iran University of Medical Science, Tehran, Iran; ^8^Department of Nursing, Alborz University of Medical Sciences, Karaj, Iran

**Keywords:** COVID-19, anxiety, perceived stress, psychological behavior response, global pandemic, COVID-19 anxiety syndrome

## Abstract

**Objective:**

Despite the abundance of studies linking fear and anxiety to COVID-19, there are limited studies that examine how these elements impact psychological behavioral responses, especially in Iran. The aim of this study was to investigate the relationship between anxiety and fear of COVID-19 with psychological behavior response, whether this relationship is mediated by role of perceived stress among Iranian population during the COVID-19 pandemic.

**Methods:**

A predictive cross-sectional study was used to investigate the relationships between COVID-19 anxiety syndrome, fear of COVID-19 with psychological behavioral responses due to the pandemic, and the mediating role of the COVID-19 perceived stress in these relationships.

**Results:**

The current study revealed that during the COVID-19 pandemic, fear and anxiety of COVID-19 can influence the psychological behavioral responses of individuals; however, this can be explained through perceived stress.

**Conclusion:**

As such, the current study points out that the individuals who perceived high stress due to COVID-19 were more likely to comply with guidelines, which has given new insight into this field. The current study findings are applicable for health policymakers in order to help them in understanding human behavior for developing health promotion programs and also for fostering resilience among the general population.

## Introduction

The outbreak of the COVID-19 virus has led to millions of deaths globally, forcing governments to take crude measures to halt the spread of the virus. The global pandemic and the subsequent public health measures taken in order to contain the virus have created a profound effect on human life, producing alarming surges in mental health problems, and economic issues (1–5). The prevalence of this virus since December 2019 has long surpassed the rates of infection and death tolls of severe acute respiratory syndrome (SARS), Middle East Respiratory Syndrome (MERS), and Ebola, causing immense psychological difficulties to the general population, which are sequelae linked to fear of infection, provoking a secondary mental health crisis (6–10).

On February 19, 2020, Iran reported its first confirmed case in the city of Qom (11), and by August 2021, the virus had infected over 4.1 million people and caused deaths of at least 94,000 Iranians, becoming the highest death toll in the Middle East (12). Due to COVID-19's alarming speed of infection worldwide, WHO declared it as a pandemic in March 2020 (13). National governments, including Iran, enforced unprecedented reforms, such as lockdowns, quarantine, closures of all non-essential business, social distancing, and intensified hygiene practices in attempt to prevent and reduce the spread of COVID-19 (14). By taking these strict measures, a consequence was the exacerbation of negative psychological responses such as anxiety, stress, uncertainty, fear, and other substantial lifestyle changes among its people (15). Studies have shown that healthcare workers (HCWs) (16), alongside adolescents (17), elderly patients (18), and people who were infected by the coronavirus, were the population hardest hit by the COVID-19 pandemic. Consequently, the COVID-19 psychological impact was observed to be prominent in HCWs (16), students, people with poor health, and women (19, 20) but were lessened when preventive health measures were taken (20), which complied with guidelines and government recommendations (21).

Evidence has shown that high levels of anxiety in Iranians can have negative effects on people's lives and can lead to serious problems (22). Anxiety due to COVID-19 has been associated with contracting and/or dying from COVID-19, fear of their families or loved ones getting infected (23, 24), financial issues (25), and fear of shortage of food, medicine, and other necessities due to panic buying and hoarding (26, 27), thus negatively affecting one's mental health (24). In the same vein, the pandemic has drastically impacted lifestyles, creating anxiety due to social connectedness (28, 29), isolation (30, 31), loneliness (32), and financial hardship (30, 33). This uncontrollable anxiety can lead to an emotional state that may overwhelm the behavior, feelings, and thoughts of the individuals, causing further mental or psychological disorders (34). In addition, obsessive thinking, and other forms of perseveration about COVID-19, may escalate the emergence of clinical anxiety and maladaptive coping (3, 35). Studies have also highlighted that the post-pandemic anxiety may be higher due to the difficulty of returning to “normal” societal functioning, which unavoidably requires exposure to environments related with a greater risk of infection, such as public transport, offices, cinemas, and theaters (35).

During the COVID-19 pandemic, fear has been the most vital and common sentiment with substantial psychological effect on individuals, imploring them to sustain sanitation during the lockdowns and quarantines, thus experiencing stress, anxiety, worry, panic, and phobia to some extent, if it is not well-calibrated (36, 37). Fear can be ascribed to the individual's knowledge of the facts related to that virus either from the media or government bodies, or by directly experiencing the illness or exposure to the indirect experience of a disease outbreak (8, 38–42). Schimmenti et al. (43) categorized fear as: fear for the body, fear for significant others, fear of not knowing, and fear of inaction and past studies have linked fear positively with increased anxiety and depressive symptoms (43–45). Parlapani et al. (19) identified women to have substantially higher levels of fear toward COVID-19 as compared to men, leading participants to have severe depressive and anxiety symptoms. In addition, they discovered that people <30 years old showed less fear of the pandemic. However, severe COVID-19 fear is linked with higher suicide risk (46, 47), psychological distress (5, 48), anxiety and depression (Ahorsu, Lin, Imani, Saffari, Griffiths, (49, 50)), xenophobia and discrimination (51, 52), and pre-existing mental health disorders (53). On the other hand, insufficient fear of the pandemic, whereby the government restrictive measures and policies to combat the pandemic are ignored (45, 54) and COVID-19 vaccine hesitancy (55), may harm the individual and society negatively.

The increasing numbers of COVID-19 infections and mortality have escalated stress (37), which is the main risk factors of mental health problems such as insomnia, anxiety, and depression (49, 54, 56, 57). Stress caused by the pandemic was found to be higher in women (9, 37, 42), younger people (58), those with poor sleeping habits (37, 58, 59), support caregivers, and other minority and disadvantaged groups (60) as they have lower compliance with prevention behavior and/or have less adaptive coping strategies, leading to substantial long-term mental health problems (61). Interestingly, people with higher education were found to have higher level of stress, anxiety, and depression during this pandemic (62), which may be due to their high self-awareness about their health (50).

Despite the abundance of studies linking fear and anxiety to COVID-19, there are limited studies, to the authors' knowledge, that examine how these elements impact psychological behavioral responses, especially in Iran. Hence, this current study was conducted to assess two research objectives, the first is to determine the effect of COVID-19 anxiety syndrome and fear of COVID-19 on psychological behavioral responses in Iran. The second objective evaluated the role of stress in mediating the relationships between COVID-19 anxiety syndrome and fear of COVID-19 on psychological behavioral responses among the Iranian people. The psychological and behavioral responses of COVID-19 in Iran is crucial to enhance resilience and to decrease the population's vulnerability.

## Methods

A predictive, cross-sectional online questionnaire-based survey was used in this study to investigate the relationships between COVID-19 anxiety syndrome, fear of COVID-19 with psychological behavioral responses due to the pandemic, and the mediating role of the COVID-19 perceived stress in these relationships.

### Participants

The requisite sample size was estimated to be 1,000, with a probability of 0.05, a statistical power of 80%, an anticipated medium effect size of 0.12, and 31 items measuring four constructs. This estimate was calculated *a priori* using a sample size calculator for Structural Equation Models (SEM) (63). The minimum statistical power analysis in humanities and social sciences studies should be 80% (64). In total, 926 participants in Iran participated between October and November 2020 during the initial stages of the COVID-19 pandemic. The online scales were created *via* Google Forms and its URL link was sent by email or social networking applications such as a Telegram channel or WhatsApp group of adults. The inclusion criteria for participants were adults (age > 18) who were willing to participate in this study. The mean age of participants was 31.12 (SD = 7.62) (range 18 to 67) years old, and most were female (85.2%), married (69.1%), and had a bachelor's degree (45.0%). Other socio-demographic information is provided in Table 1.

**Table 1:** Demographic characteristics of participants (*n* = 926).

**Variables**	***n*** **(%)**
**Gender**	
Female	782 (85.2)
Male	137 (14.8)
**Marital status**	
Single	286 (30.9)
Married	640 (69.1)
**Education level**	
Under diploma	19 (2.1)
Diploma	128 (13.8)
Upper Diploma	58 (6.3)
Bachelor	417 (45.0)
Master	243 (26.2)
Ph.D.	54 (5.8)

### Instruments

A demographic form and the Persian version of the following scales were used in this study.

### Perceived Stress Scale

The PSS-10 is a self-reported scale to measure the global level of perceived stress (65). This scale includes two factors: Factor 1 (Perceived Helplessness) is made of negatively phrased items (i.e., items 1, 2, 3, 6, 9, and 10; e.g., “In the last month, how often have you felt nervous and stressed?”), and Factor 2 (Perceived Self-Efficacy) is made of positively phrased items (i.e., items 4, 5, 7, and 8; e.g., “In the last month, how often have you felt that things were going your way?”).

### The Persian Version of the COVID-19 Anxiety Syndrome

This self-report measure includes nine items, loading on two factors, assessing features of the anxiety syndrome linked to COVID-19. These are (1) avoidance (e.g., of public transport because of the fear of contracting COVID-19); (2) checking (e.g., of symptoms of COVID-19); (3) worrying (e.g., researching symptoms of COVID-19 at the cost of other activities); and (4) threat monitoring (e.g., paying close attention to others displaying possible symptoms of COVID-19. Items relating to checking, worrying, and threat monitoring load on the first factor (“perseveration”) with a second factor comprising avoidance items (“avoidance”). Participants are asked to rate how frequently they experience each feature of the anxiety syndrome using a 5-point time anchored scale (0 = “Not at all” to 4 = “Nearly every day over the last 2 weeks”). Scores range between 0 and 36, with higher scores indicative of increased levels of the anxiety syndrome. The C-19ASS has demonstrated good reliability and validity (35). In the current study, the Cronbach α was 0.82.

### The Persian Version of the Fear of COVID-19

The FCV-19S (44) is a seven-item scale that assesses the fear of COVID-19. The seven items (e.g., “I am most afraid of coronavirus-19”) are rated on a 5-point scale from 1 (strongly disagree) to 5 (strongly agree) with scores ranging from 7 to 35. The higher the score, the greater the fear of COVID-19.

### The Persian Version of the Psychological Behavioral Responses

The PBR (66) is a self-reported measure that assesses the characteristics of psychological and behavioral responses in COVID-19. This measure includes 5 items with scores ranging from 1 (never) to 4 (always) and has good validity and reliability.

### Data Analysis

To assess factor structure, exploratory factors analysis (EFA) was performed through maximum likelihood with Promax rotation using SPSS version 26. The Kaiser–Meyer–Olkin (KMO) and the Bartlett's test of sphericity were employed to ensure the study sample was appropriate to perform the factor analysis. Items with absolute loading below 0.5 were removed (67). Next, following the two-step approach, this study employed covariance-based structural equation modeling and Amos version 27 to test the measurement model and structural model. First, to assess the measurement model, the maximum likelihood confirmatory factor analysis (CFA) was performed. Model fit was assessed using several model fit indexes and the model was revised according to the modification indices (67). The internal consistency of each construct was assessed using its Cronbach's alpha. Construct reliability was assessed using composite reliability (CR) and maximal reliability (MaxR). The convergent validity was assessed through average variance extracted (AVE) of the latent constructs. Cronbach's alpha, CR, and MaxR were >0.7, indicating good internal consistency and construct reliability, while AVE of >0.5 indicates good convergent validity. To establish discriminant validity, the heterotrait–monotrait ratio of correlation (HTMT) matrix with values <0.85 was considered acceptable discriminant validity (68). Next, the proposed model and hypothesis were tested. In order to test the hypotheses in the structural model, bootstrapping with 2,000 replications was performed (67). All tests in this study were two-tailed, and *p* values of <0.05 were considered statistically significant.

### Ethical Considerations

The study aims, number of items, time to complete the survey, the researchers' affiliation and email for queries, and the ethical code of study were inserted on the first page of the online questionnaire. These items informed participants that their participation was voluntary and that their responses would be published anonymously as group data. The protocol of this study was approved by the Mazandaran University of Medical Sciences Research Ethics Committee (IR.MAZUMS.REC.1400.13728).

## Results

The results of the maximum likelihood EFA with Promax rotation extracted five factors, in which COVID-19 anxiety syndrome was divided into two factors, namely, perseverate thinking (five items) and avoidance (four items). The values of Kaiser–Meyer–Olkin (KMO) was 0.911 and Bartlett's test of sphericity showed the adequacy of the sampling and suitability of the data for performing the factor analysis (*p* < 0.001, χ^2^ = 10,557.720, df = 300). One item from perceived stress and two items from psychological behavioral responses were removed due to weak factor loadings of <0.5. The final factor structure explained 57.793% of the variance.

The maximum likelihood CFA was performed to assess the measurement model based on the factor structure obtained from EFA. The results showed that the initial measurement model with all first-order construct did not fit the data well [χ^2^(242) = 1,283.852, *p* < 0.001, χ^2^/*df* = 5.305, CFI = 0.898, IFI = 0.898, TLI = 0.883, SRMR = 0.059, and RMSEA (90% CI) = 0.068 (0.065, 0.072)]. Following the results of modification indices, five pairs of the item measurement error (i.e., anxiety syndrome—two pairs; fear of COVID-19—three pairs) were allowed to freely covary to improve the model fit. The revised measurement model with all first-order constructs has improved significantly [Δχ^2^ (Δ*df* = 4) = 411.581, *p* < 0.001] and fitted the data well [χ^2^(238) = 872.271, *p* < 0.001, χ^2^/*df* = 3.665, CFI = 0.938, IFI = 0.938, TLI = 0.928, SRMR = 0.053, and RMSEA (90% CI) = 0.054 (0.050–0.058)]. Next, COVID-19 anxiety syndrome was included in the revised measurement model as second-order construct, and the results showed that the final measurement model fit also fitted the data well [χ^2^(240) = 1,016.966, *p* < 0.001, χ^2^/*df* = 4.237, CFI = 0.924, IFI = 0.924, TLI = 0.912, SRMR = 0.053, and RMSEA (90% CI) = 0.059 (0.055, 0.063)], and all factor loadings were >0.5 and significantly.

Table 2 shows the results of the measurement model assessment. All constructs (both first-order and second-order constructs) showed good internal consistency (Cronbach's alpha ranged from 0.721 to 0.886), and construct reliability (CR ranged from 0.724 to 0.876, MaxR ranged from 0.732 to 0.889). As shown in Table 2, all constructs' AVE was >0.5, except for construct of avoidance (0.398) and psychological behavioral responses (0.471). Although the AVE for these two constructs was <0.5, Fornell and Larcker (69) recommended that if AVE is <0.5, CR of >0.7 alone can be used to establish convergent validity of the construct. Indeed, AVE is a strict measure of convergent validity and a more conservative measure than CR (70). Therefore, all constructs have achieved convergent validity.

**Table 2:** Results of the Measurement model assessment.

**Construct**	**Factor loading**	**Cronbach's alpha**	**CR**	**MaxR**	**AVE**
**First order construct**					
**Perseverate thinking**					
Item 1	0.659	0.846	0.844	0.860	0.524
Item 2	0.588				
Item 3	0.814				
Item 4	0.825				
Item 5	0.706				
**Avoidance**					
Item 1	0.538	0.721	0.724	0.732	0.398
Item 2	0.684				
Item 3	0.671				
Item 4	0.618				
**Fear of COVID-19**					
Item 1	0.758	0.886	0.876	0.889	0.506
Item 2	0.813				
Item 3	0.611				
Item 4	0.740				
Item 5	0.793				
Item 6	0.538				
Item 7	0.678				
**Perceived Stress**					
Item 1	0.722	0.863	0.863	0.868	0.559
Item 2	0.789				
Item 3	0.792				
Item 5	0.683				
Item 6	0.746				
**Psychological Behavioral Responses**					
Item 3	0.546	0.721	0.724	0.755	0.471
Item 4	0.798				
Item 5	0.691				
**Second order construct**					
COVID-19 Anxiety Syndrome		0.830	0.749	0.895	0.559
Perseverate thinking	0.578				
Avoidance	0.943				

Table 3 shows the results of HTMT matrix, and all values in the HTMT matrix were <0.9, demonstrating the acceptable discriminant validity of all constructs.

**Table 3:** Discriminant validity assessment using HTMT matrix.

		**(1)**	**(2)**	**(3)**	**(4)**	**(5)**
Heterotrait-monotrait ratio of correlations (HTMT)	**First order construct**					
	Perseverate thinking					
	Avoidance	0.552				
	Fear of COVID-19	0.615	0.482			
	Perceived Stress	0.343	0.237	0.505		
	Psychological Behavioral Responses	0.252	0.834	0.306	0.065	
	**Second order construct**					
	(6) COVID-19 Anxiety Syndrome			0.645	0.344	0.564

Next, the proposed structural model and hypotheses were tested while controlling for the effect of participants' age, gender, marital status, and education level. The results of the structural model assessment are shown in Table 4. The results of assessing total effect showed a significant positive relationship between COVID-19 anxiety syndrome and psychological behavioral responses (*b* = 0.767, *p* < 0.001), and between fear of COVID-19 and psychological behavioral responses (*b* = 0.121, *p* < 0.001), providing support for H1 and H2. The total effect model explained 68% of the total variance of psychological behavioral responses. Moreover, the results of assessing direct effect showed a significant positive relationship between COVID-19 anxiety syndrome and perceived stress (*b* = 0.113, *p* < 0.01), between fear of COVID-19 and perceived stress (*b* = 0.455, *p* < 0.001), and between perceived stress and psychological behavioral responses (*b* = 0.100, *p* < 0.001); thus, H3, H4, and H5 were supported. Lastly, using a bootstrapping approach, the results of assessing indirect effects supported H6 and H7 on the positive mediation role of perceived stress in the relationship between COVID-19 anxiety syndrome and psychological behavioral responses (*b* = 0.011, *p* < 0.01) and between fear of COVID-19 and psychological behavioral responses (*b* = 0.046, *p* < 0.001). The significant direct relationship between COVID-19 anxiety syndrome and psychological behavioral responses (*b* = 0.756, *p* < 0.001) and between fear of COVID-19 and psychological behavioral responses (*b* = 0.075, *p* < 0.01) indicates that the mediation role of perceived stress for both relationships was partial.

**Table 4:** Structural model assessment.

**Paths**	**Unstandardized Path**	**95% confidence level**
	**coefficients**	**(Lower Bound, Upper Bound)**
**Total Effect**		
COVID-19 Anxiety Syndrome → Psychological Behavioral Responses	0.767^***^	(0.708,0.832)
Fear of COVID-19 → Psychological Behavioral Responses	0.121^***^	(0.089,0.155)
**Direct Effects**		
COVID-19 Anxiety Syndrome → Perceived Stress	0.113^**^	(0.043,0.188)
Fear of COVID-19 → Perceived Stress	0.455^***^	(0.412,0.496)
Perceived Stress → Psychological Behavioral Responses	0.100^***^	(0.074,0.127)
COVID-19 Anxiety Syndrome → Psychological Behavioral Responses	0.756^***^	(0.698,0.820)
Fear of COVID-19 → Psychological Behavioral Responses	0.075^**^	(0.041,0.112)
**Mediation Effects**		
COVID-19 Anxiety Syndrome → Perceived Stress → Psychological Behavioral Responses	0.011^**^	(0.004,0.021)
Fear of COVID-19 → Perceived Stress → Psychological Behavioral Responses	0.046^***^	(0.033,0.060)

*** *p < 0.001*,

** *p < 0.05; Control variables: age, gender, marital status, and education level*.

**Figure 1:**
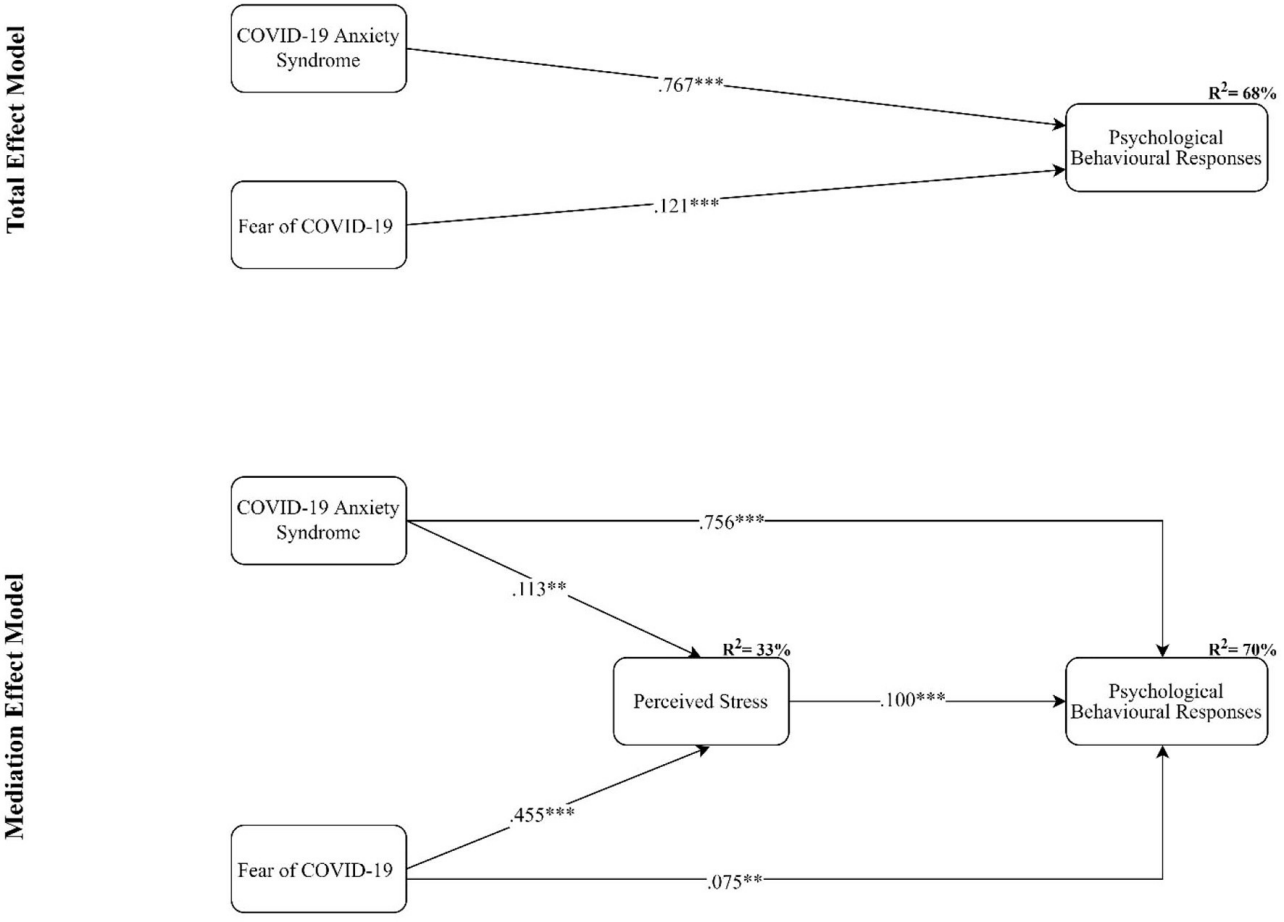
The results of the structural model assessment; ***p < 0.001, **p < 0.005; Control variables: age, gender, marital status, and education level.

The mediation model explained 70% of the total variance of psychological behavioral responses and 33% of the total variance of perceived stress. Figure 1 shows the results of the structural model.

## Discussion

The current study sought to assess the relationship between COVID-19 anxiety syndrome and fear of COVID-19 with psychological behavioral responses. Moreover, the study aimed to examine whether the COVID-19 perceived stress mediates the relationship between COVID-19 anxiety syndrome, fear of COVID-19, and psychological behavioral responses.

The findings revealed that there was a significant positive correlation between COVID-19 anxiety syndrome and psychological behavioral responses. This finding aligns with previous studies in determining a positive relationship between the COVID-19 anxiety syndrome and psychological behavioral responses such as depression, feelings of helplessness, persistent worrying, and never feeling clean after disinfecting (71). Pandemic psychological distress can shape the behavior (35) and it has been identified that people usually experience fear, sense of isolation (72), and a wide range of behavioral change (73) during novel pandemics (74). However, in response to the stress experienced by people as a result of COVID-19, there are many behavioral changes that have led to over-compliance with health protocols as well as many reports of non-compliance with these protocols such as wearing masks and hand washing (39). The current article has identified that a person's gender, age, and educational level have increased the likelihood of non-compliance with COVID-19-related public health measures. The findings of a longitudinal cohort study have indicated that non-compliance, especially with hygiene-related measures, was more prevalent in male participants and individuals with higher educations (75). This is why we have controlled the effects of gender, age, and education during the data analysis.

The current study also explored the positive correlation between fear of COVID-19 and psychological behavioral responses. According to the protection motivation theory (PMT), which proposed key contributors to people's willingness to make behavioral changes (76), the extent of the fear that the individual perceives, as well as the other factors such as coping skills, have the potential to determine individuals' behavioral response. The COVID-19 pandemic formed several fears for people such as fear of being contaminated (72) or the fear of the unknown (77, 78) that can trigger elements related to psychological behavioral responses. Due to the novel nature of the current pandemic with a rapid person-to-person transmission, as well as its potential for transmission from asymptomatic carriers, individuals may experience a threat that causes fear (19, 79). Fear of COVID-19 can lead to protective behaviors (49). It has been revealed by research (54) that fear of COVID-19 was the only predictor of positive behavior change such as improved hand hygiene or social distancing. Interestingly, they found that the COVID-19 fear and anxiety were stronger predictors than moral and political orientation (54). Similarly, fear can significantly increase individual engagement in preventive behaviors during the COVID-19 pandemic (80). It is worth mentioning that the relationship between fear and health behaviors is 2-fold. A study conducted in Greece (2020) among 3,029 participants indicated that the greater application of safety or checking behaviors, as well as a high level of compliance with guidelines, led to an amplification of fear, potentially due to increased contamination awareness (19).

It has been suggested by the current study that there is a significant positive relationship between COVID-19 anxiety syndrome and perceived stress. Also, the significant positive correlation between fear of COVID-19 and perceived stress was shown by the current study's findings. The person's appraisal of a stressor as threatening or not, as well as her/his own abilities to cope can indicate the perceived stress level (81). Several factors such as the inconsistency between policies and scientific evidence (82), the lockdown policies and quarantine (83, 84), evidences of possible fatal consequences of contracting the virus (85), repeated exposure to media reports (52), and the individual psychological trait (86) influence the perceived stress associated with COVID-19. The findings of an Iranian study have indicated that the most stressful event during the COVID-19 outbreak was the rise in essential goods prices. They have also found that the death of a family member due to COVID-19 infection was the main source of perceived stress (87). The anxiety and fear of contracting COVID-19 are also identified as the most important underlying factor influencing the level of COVID-19 perceived stress. The findings of a study (88) showed that higher COVID-19 perceived stress was associated with more emotional distress including fear and anxiety. It has been indicated that perceived stress due to COVID-19 among the Iranian general population was slightly high, and it has been correlated with using social media (89). Previous studies have also shown that, in some cases, social media can increase the perceived risk of the outbreaks (90). The findings of a large national study in Iran found a high level of stress among the general Iranian population during the COVID-19 outbreak in which those in middle age groups and low to moderate socioeconomic status experienced the highest stress due to worry about losing their jobs or income (91).

The findings of the current study showed that there is a positive significant correlation between the perceived stress of COVID-19 and psychological behavioral responses. Furthermore, the perceived stress of COVID-19 mediated the relationship between COVID-19 anxiety and fear of COVID-19, and psychological behavioral responses. Although there are studies that indicate that the more the individual perceives the stress, the higher the potential for engaging in unhealthy behaviors (92), the current study showed a contradictory finding. As such, the current study points out that the individuals who perceived high stress due to COVID-19 were more likely to comply with guidelines. This finding is supported by previous findings that indicated that practicing precautionary behaviors during the COVID-19 pandemic is strongly associated with perceived stress (66, 93). Some existing studies addressed the mediating role of perceived stress in relationships between different concepts and psychological behavioral responses in different settings. For example, a study conducted by Pfeffer et al. (94) indicated the moderating role of perceived stress and trait self-control in the context of intention and physical activity behavior. It has also been found that nearly half of the total effect of self-compassion on health behavior occurred through perceived stress (95). According to the transactional stress model (96), individuals' reactions and adaptation to the objective stressful events are determined by their cognitive appraisal of the stressors such as perceived stress. It has been addressed by the studies' findings that those individuals who perceive the high levels of stress may have more difficulty in realizing positive cognition, emotion, and behaviors and are at a greater risk for health problems (88, 97). However, the current study indicated that the more individuals perceived the COVID-19 stress, the higher the compliance with the protective measures. In line with this finding, a cross-sectional study with 3,727 Iranian participants revealed that respondents were motivated by the COVID-19 danger and fear control responses that indicates their high perceived efficacy (98). The extended parallel process model (EPPM) (99) suggests supporting theoretical explanation for the current study finding. EPPM suggests that individuals who are exposed to a risky situation usually apply two types of cognitive appraisal, namely, the efficacy of the recommended advice and perceived threat. Accordingly, individuals who perceive the COVID-19 threat in high levels while perceiving low efficacy usually act to protect themselves from the fear rather than the danger itself (fear control process). Instead, those who perceive high efficacy, even if they perceive a high level of threat, usually will be motivated to protect themselves from the danger (danger control process).

### Study Limitations

While the study provides new information relative to the mediating role of the perceived stress on the relationship between COVID-19 anxiety syndrome, fear of COVID-19, and psychological behavioral responses, it is not without its limitations. The cross-sectional design of this study does not allow for firm causal conclusions. Conducting longitudinal studies by collecting data at different points in time as well as experimental studies are recommended for future research since there are numerous complex and dynamic processes by which spirituality relates to mental health outcomes. In terms of mediation studies, the most salient mediating processes seem to involve stress dimensions, values/attitudes, and social control/norms, which need to be investigated in further studies. Furthermore, the data were gathered *via* online data collection. Despite its advantages (e.g., affordability and accessibility), online surveys have been criticized for selection bias and difficulty reaching certain types of participants (100, 101).

## Conclusion

The current study revealed that during the COVID-19 pandemic, fear and anxiety of COVID-19 can influence the psychological behavioral responses of the individuals; however, this can be explained through perceived stress. The visibility of protective factors in addition to risk factors can offer a broader view on measures to deal with depression in the general population resulting from global adverse situations such as the ongoing COVID-19 pandemic. The current study findings are applicable for health policymakers to help them in developing health promotion programs and fostering resilience among the general population. Also, it is useful for organizations and workplaces because they have been known as the best place to provide psychological support to the general population. Workplaces have a considerable role in preventing the spread of COVID-19 infection, and conducting health promotion programs to increase psychological skills and coping mechanisms to address the negative effects of the COVID-19 pandemic (102).

## Data Availability Statement

The original contributions presented in the study are included in the article/supplementary material, further inquiries can be directed to the corresponding author/s.

## Ethics Statement

The studies involving human participants were reviewed and approved by Mazandaran University of Medical Sciences Research Ethics Committee (IR.MAZUMS.REC.1400.13728). Written informed consent to participate in this study was provided by the participants' legal guardian/next of kin.

## Author Contributions

All authors listed have made a substantial, direct, and intellectual contribution to the work and approved it for publication.

## Conflict of Interest

The authors declare that the research was conducted in the absence of any commercial or financial relationships that could be construed as a potential conflict of interest.

## Publisher's Note

All claims expressed in this article are solely those of the authors and do not necessarily represent those of their affiliated organizations, or those of the publisher, the editors and the reviewers. Any product that may be evaluated in this article, or claim that may be made by its manufacturer, is not guaranteed or endorsed by the publisher.
